# Identifying Driving Forces of Built-Up Land Expansion Based on the Geographical Detector: A Case Study of Pearl River Delta Urban Agglomeration

**DOI:** 10.3390/ijerph17051759

**Published:** 2020-03-08

**Authors:** Yongwei Liu, Xiaoshu Cao, Tao Li

**Affiliations:** 1 School of Business, Ludong University, Yantai 264025, China; 2 Institute of Transport Geography and Spatial Planning, Shaanxi Normal University, Xi’an 710119, China; caoxsh@mail.sysu.edu.cn (X.C.); ltao@snnu.edu.cn (T.L.); 3 School of Geography Science and Planning, Sun Yat-sen University, Guangzhou 510275, China

**Keywords:** built-up land expansion, landscape metrics, spatial autocorrelation, geographical detector, pearl river delta urban agglomeration

## Abstract

Understanding the driving forces behind built-up land expansion is crucial in urban planning and management. Using the Pearl River Delta urban agglomeration as research area, four landscape metrics were used to analyze landscape characteristics of urban expansion from 1990 to 2015. Spatial autocorrelation analysis was used to study the characteristics of built-up land expansion, while geographical detector was employed to identify the driving forces of urban land growth and their interactions. The results show the extent of built-up land has been increasing, the structure has become more complex, the level of fragmentation has been increasing, and the aggregation degree is in decline. The built-up landscape index shows spatial heterogeneity occurring in the core and peripheral towns of cities, as well as in the core and peripheral areas of the entire region. Also, changes in the built-up landscape index indicate increased spatial aggregation occurring in the past 25 years. Results from the geographical detector show natural, socio-economic, and transportation-related factors have substantial influence on built-up land expansion. Elevation, slope, population density, change in population density, and road network density were shown to have high influencing power. The influencing powers of slope and change in population density were also found to be different from other factors, highlighting their important role in urban development. Also, there were two types of interactions found, enhance nonlinear and enhance bivariate interactions, indicating the compounding influence of interactions between significant determinants. This study provides a new perspective and methodological approach in evaluating the driving forces behind built-up land expansion and their interactions.

## 1. Introduction

Urbanization has become a global phenomenon, which has significantly transformed society and the global economy and has become a crucial geospatial process [1]. With urbanization, research on urban transport [2], spatial planning [3] and smart city [4] has been increasing. Over the past 30 years, urban populations have increased, and built-up land has expanded rapidly, especially in developing countries [5]. Rapid urbanization has had profound influence on the structure and functions of both natural ecosystems and human livelihoods [6,7] and has resulted in various problems, such as ecological destruction, resource shortages, population explosion, environmental pressure, and health problems [8,9,10,11,12,13]. Understanding the process of built-up expansion and its driving factors is crucial for effective urban growth planning and management to mitigate associated adverse impacts [14].

In order to investigate the effects of various factors on built-up land expansion, numerous models and approaches have been used, including cellular automata [15,16], bivariate regression [17,18], analytical hierarchical process [19], logistical regression [20,21], and spatial regression [5,22]. Previous studies have tried to establish how landscape characteristics and determinants of built-up land expansion, such as shape, fragmentation, and edge, provide an efficient approach to describe urban processes and their consequences [5].

Understanding the interactions among the various driving factors is crucial in accurately simulating and predicting built-up land expansion patterns; however, only a few of the methods previously stated are able to evaluate interaction effects [23]. As a spatial statistical method, the geographical detector can be used to assess the relationships of different geographical strata and identify associations between complex sets of factors and numerous geographical phenomena without using assumptions or restrictions [24]. It can quantitatively characterize the interactions between pairs of factors and obtain valuable results [23,25]. Geographical detector is a tool for measure of spatial stratified heterogeneity and attribution of spatial patterns, the factor detector, interaction detector and ecological detector can analyze interactions between two factors, excellent ability to study the factor interactions. Thus, with the use of the geographical detector, the spatial pattern of built-up land expansion can be better understood, and the interactions between driving forces and their relation to urban growth can be quantitatively characterized. Various factors can influence the expansion of built-up lands. After an extensive literature review, we found that several variables have often been considered in past studies. These include topographic factors [5,14,23,26,27,28,29,30,31,32], population [18,23,26,29,30,31,32,33,34], Gross Domestic Product (GDP) [18,23,26,30,33,35,36], road effects [5,14,23,28,29,31,34], policy factors [21,28,29,30,31,34], and other factors [37,38,39].In this study, we explore the attributes and the impact of built-up land expansion and characterize the interactions between these factors. In particular, we address the following research questions: (1) What are the landscape pattern characteristics of built-up land expansion? (2) What are the features of the impact of driving forces of built-up land expansion and the interactions between these factors? Using geographic information systems (GIS) and remote sensing technology, we employed geographical detector and landscape metrics to explore the landscape characteristics and impact of built-up land expansion for the Pearl River Delta (PRD) urban agglomeration from 1990 to 2015.

## 2. Materials and Methods 

### 2.1. Study Area

**Figure 1:**
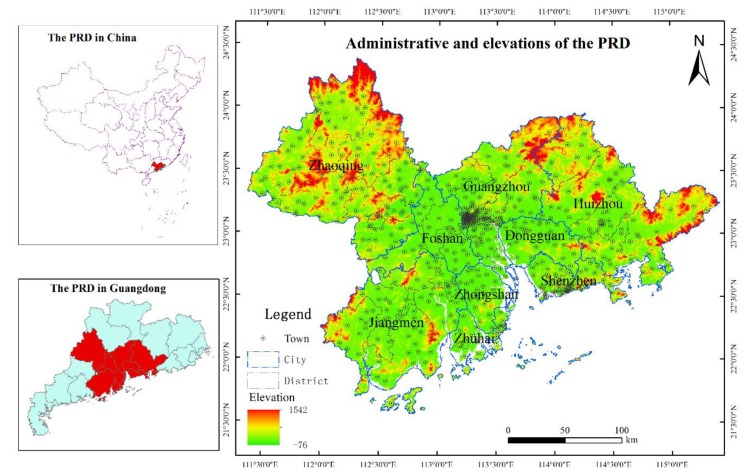
The location of the Pearl River Delta urban agglomeration.

Adjacent to the South China Sea, the PRD is located in southern mainland China and is one of the three most economically developed regions in China. The region includes two deputy provincial cities, Guangzhou and Shenzhen, and seven prefecture-level cities, namely Foshan, Dongguan, Zhuhai, Huizhou, Zhongshan, Jiangmen, and Zhaoqing [40]. With continued economic progress in PRD, urban construction has accelerated significantly in recent years. In 2015, the area of built-up land was estimated at about 9127 km^2^, which constituted approximately 17% of the total land area. Urbanization was particularly pronounced in Shenzhen, Dongguan, Zhongshan, and Foshan, making more than 30%. In this study, 581 towns situated in the Pearl River Delta urban agglomeration were selected as the research area. The location map of the study area is shown in Figure 1.

### 2.2. Data Source and Description

The built-up land areas were derived using Landsat images: Landsat-5 TM for 1990 and Landsat-8 OIL (National Aeronautics and Space Administration, Washington, DC, USA) for 2015. All Landsat images were classification by supervised classification technique. The resulting classification map delineated built-up lands against non-built-up lands, supervised classification accuracies of the 1990 and 2015 were 89.21% and 93.12%, having higher reliability.

Advanced Spaceborne Thermal Emission and Reflection Radiometer Global Digital Elevation Model (ASTER GDEM, version 2) (National Aeronautics and Space Administration, Washington, DC, USA) was used as the elevation data source, and the slope was obtained by processing the ASTER GDEM data using ArcGIS 10.2 software (Environmental Systems Research Institute, Redlands, CA, USA). The population and gross domestic product (GDP) data for 1990 and 2015 were obtained from the Guangdong Statistical Yearbook of 1991 and 2016. The road data were acquired from the Guangdong Transportation Atlas of 1992 and 2016 and were digitized as vector road data using ArcGIS.

### 2.3. Methods

#### 2.3.1. Spatial Autocorrelation Analysis

Spatial autocorrelation analysis has been widely used in land use and landscape studies [22,41,42,43] and utilizes global spatial autocorrelation index and local spatial autocorrelation index. Moran’s I is used to measure global spatial autocorrelation, the formula as follows:

(1) $$I=\frac{n\sum_{i}^{n}\sum_{j}^{n}w_{ij}\left(y_{i}-\bar{y}\right)\left(y_{j}-\bar{y}\right)}{\left(\sum_{i}^{n}\sum_{j}^{n}w_{ij}\right)\sum_{i}^{n}{\left(y_{i}-\bar{y}\right)}^{2}}$$

where $y_{i}$ denotes the values of points *i*, $y_{j}$ denotes the values of points *j*, $\bar{y}$ is the average of all values, $w_{ij}$ is the spatial weight, and *n* is the total number of units. Moran’s *I* ranges from [–1, 1], which indicates a negative correlation when Moran’s *I* is less than 0, an independent random distribution when Moran’s I equal to 0, and a positive correlation when Moran’s *I* is greater than 0.

The local Moran’s *I* ($I_{L})$ is used to measure local spatial autocorrelation [44], the formula as follows:

(2) $$I_{L}=z_{i}\underset{i}{\sum }w_{ij}z_{j}$$

where $z_{i}$ is the standard amount of mean value, $z_{j}$ is the standardized quantity of the standard deviation, $z_{i}=\frac{x_{i}-\bar{x}}{\delta }$, $x_{i}$ denotes the values of points i, δ is the standard deviation of $x_{i}$. 

#### 2.3.2. Geographical Detector

The geographical detector is a spatial statistical method used to test the relationships between geographical phenomena and their potential driving factors [45,46]. It has been applied to various research themes, such as ecological and landscape connectivity [25], environmental risks [24], and built-up land expansion [23]. The geographical detector includes a factor detector, an interaction detector, an ecological detector, and a risk detector. The Geodetector software (Institute of Geographic Sciences and Natural Resources Research, Beijing, China) (http://www.geodetector.cn/) was developed to calculate the result, the tools are free of charge, freely downloadable, and easy to use, and were designed without any GIS plug-in components and with “one click” execution. The principle behind the geographical detector is that variable *Y* is associated with variable *X* if their spatial distributions tend to be identical. The Power of q value measures the association between *Y* and *X*, which is expressed as:

(3) $$q=1-\frac{1}{N\sigma^{2}}\overset{L}{\underset{h=1}{\sum }}N_{h}\sigma_{h}^{2}$$

where *N* is the number of samples, *L* is for the number of the sub-areas (*h* = 1, 2, …, L), $\sigma^{2}$ is the global variance, $\sigma_{h}^{2}$ is the variance of the sub-areas. The spatial stratified heterogeneity *q* ranges [0, 1], such that *q* = 0 indicates that *Y* is not spatially stratified heterogeneously, or simply that there is no association between *Y* and X. If *q* = 1, *Y* is said to have perfect spatial stratified heterogeneity, or simply that *Y* is entirely determined by X. The value of q indicates the degree of spatial stratified heterogeneity of *Y*, or how much *Y* can be interpreted by *X* [45,46].

#### 2.3.3. Quantifying the Built-Up Land Expansion Pattern

A variety of landscape metrics have been proposed and applied to quantify different spatial characteristics of built-up land areas, such as fragmentation [47], shape complexity [48], heterogeneity [49], and urban land patterns [16,48]. In this study, we chose four landscape metrics to characterize the built-up land expansion pattern, namely the percentage of landscape (PLAND), edge density (ED), aggregation index (AI), and perimeter-area ratio_mean (PARA_MN). The description of the landscape metrics is presented in Table 1. These four metrics are able to adequately characterize land expansion patterns and structures, and their numerical values were calculated using FRAGSTATS 4.2 software (University of Massachusetts in Amherst, Amherst, MA, USA). The change in landscape metric can, using the equation:

(4) $$C=\left(L_{2015}-L_{1990}\right)\times 100\%$$

where C stands for the change of built-up landscape metrics (i.e., $C_{PLAND}$, $C_{ED}$, $C_{AI}$, and $C_{PARA\_MN}$); $L_{1990}$ is for the value of the landscape metrics in 1990 (i.e., $L_{1990-PLAND}$, $L_{1990-ED}$, $L_{1990-AI}$, and $L_{1990-PARA\_MN}$); $L_{2015}$ is for the value of the landscape metrics in 2015 (i.e., $L_{2015-PLAND}$, $L_{2015-ED}$, $L_{2015-AI}$ and $L_{2015-PARA\_MN}$).

## 3. Results and Discussion

### 3.1. Landscape Patterns of Built-Up Land Expansion

#### 3.1.1. Spatial Patterns of Built-Up Landscape Changes

**Figure 2:**
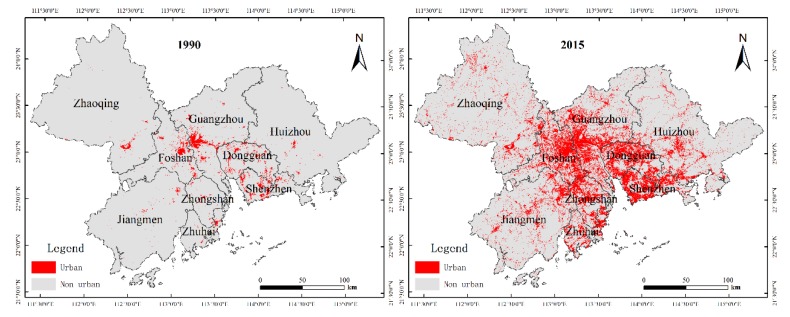
Maps of the built-up land in 1990 and 2015.

As shown in Figure 2, the area of built-up land in PRD had increased rapidly from 1202.87 km^2^ in 1990 to 9127.09 km^2^ in 2015. The growth in built-up land by 7924.22 km^2^ in 25 years means that the average annual growth rate was at 26.35%.

The summary of the results of the built-up landscape analysis using FRAGSTATS4.2 (University of Massachusetts in Amherst, Amherst, MA, USA) is shown in Table 2. The percentage of built-up land (PLAND) increased from 2.21 in 1990 to 16.69 in 2015, indicating continued urbanization in the study area. Edge density (ED) surged from 2.31 in 1990 to 31.07 in 2015, indicates the ratio of the total edge length of all built-up land patches over the total landscape area have substantially improved, mean that the level of fragmentation have improved from 1990 to 2015. which suggests that the degree of fragmentation have substantially improved. The perimeter-area ratio_mean (PARA_MN), which is indicative of shape complexity, also increased in value, suggesting that urban spread has become more complex. In terms of aggregation index (AI), the value decreased marginally from 91.37 in 1990 to 86.04 in 2015, which implies slight changes have occurred on the aggregated distribution of built-up lands. In summary, at the landscape-level, the extent of built-up land had increased significantly, fragmentation intensified, distribution characteristics became more complex, while the degree of aggregation declined.

**Figure 3:**
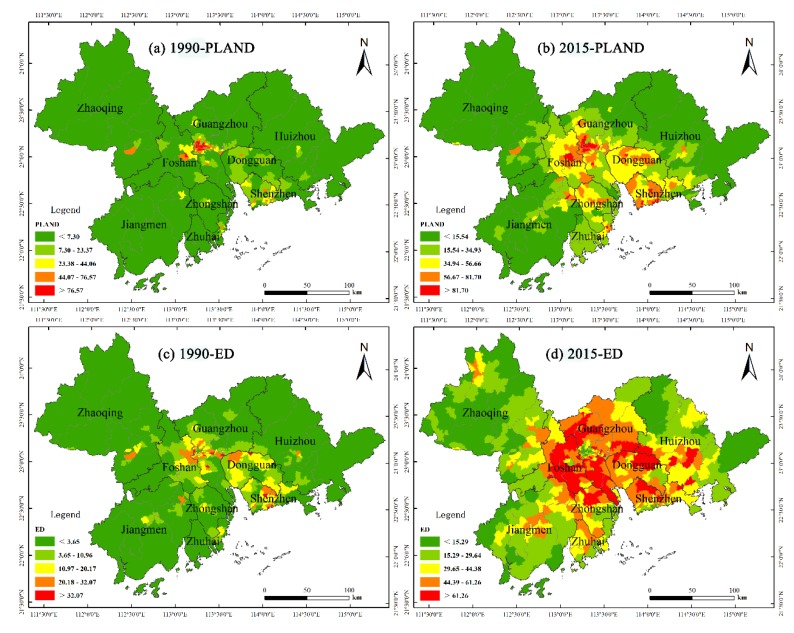

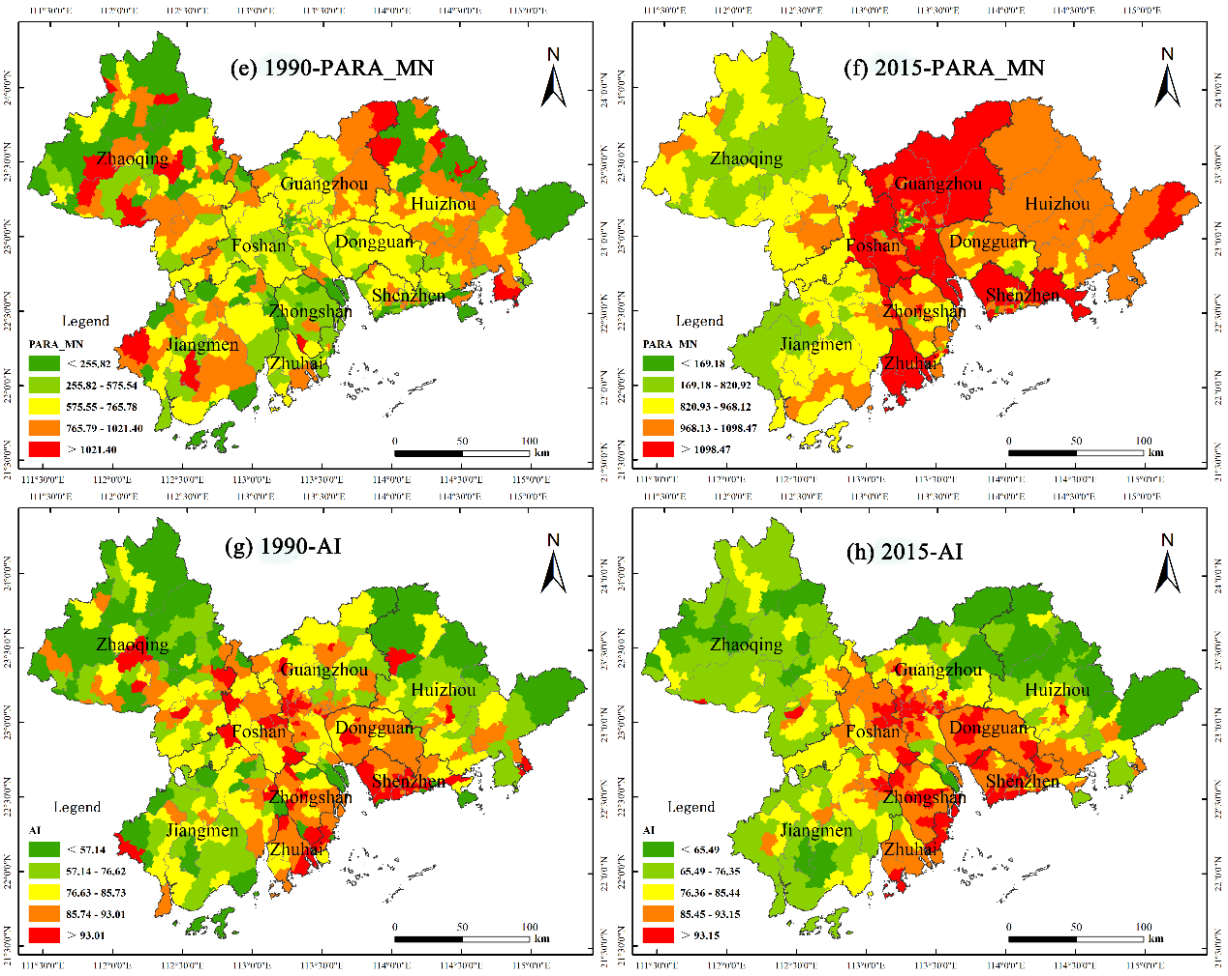
Maps of the built-up landscape metric in 1990 and 2015.

The landscape metrics of the 581 towns in PRD were calculated at the class-level (a specific type of land use) for 1990 and 2015, and the results are presented in Figure 3. In 1990 (see Figure 3a), built-up land patches are mainly located in the central towns of large, populated cities, such as Guangzhou and Foshan. In 2015 (see Figure 3b), areas with high proportions of built-up land increased significantly and extended to other cities, such as Dongguan, Shenzhen, and Zhongshan. In terms of edge density, the areas with high edge density values in 1990 (Figure 3c) were limited and were concentrated mainly in small towns in Guangzhou, Foshan, Dongguan, Shenzhen, and Zhuhai. In 2015, the spatial concentration demonstrated became more evident, with areas having high edge density values expanding towards the periphery (see Figure 3d). Thus, a distribution trend of edge values becomes more evident wherein high values are concentrated in core areas and gradually decrease into the margins. In terms of the perimeter-area ratio_mean, the dispersion was more widespread, and there were no clear town having extremely high values for 1990 (Figure 3e). For 2015, the areas with high mean ratio values significantly proliferated and shifted towards the central areas of PRD and can be found mainly in the cities of Guangzhou, Foshan, Zhuhai, and Shenzhen (see Figure 3f). A distribution pattern has formed where the values at the eastern bank of the Pearl River Estuary are considerably larger than those in the western bank. In terms of aggregation index, there are no observable regular distribution features found for 1990 (Figure 3g); but in general, the high values can be found in the city center. For 2015, the values are shown to gradually decrease from the core areas on both sides of the Pearl River Estuary towards the peripheral areas (Figure 3h).

**Figure 4:**
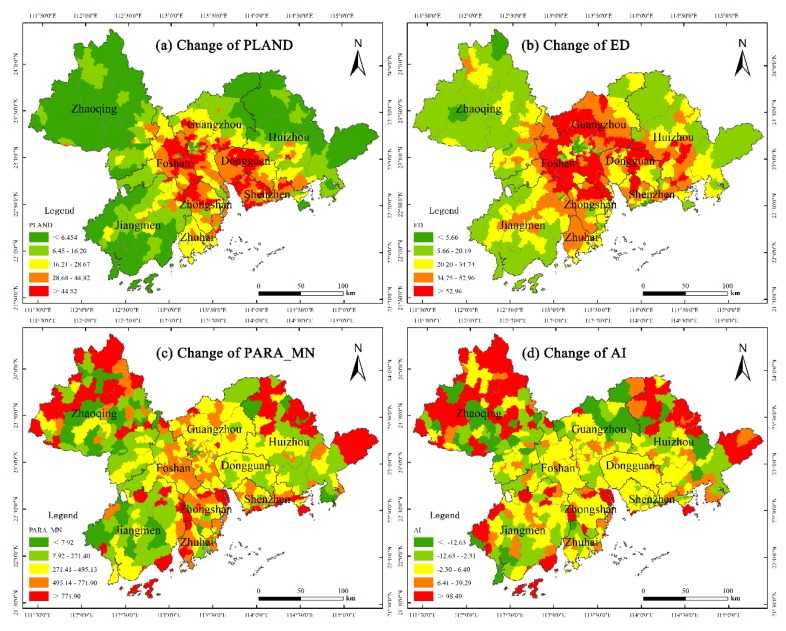
Maps of the $C_{PLAND}$, $C_{ED}$, $C_{AI}$, and $C_{PARA\_MN}$.

To further evaluate the changes in landscape patterns occurring in the cities, the spatial distribution diagrams of the landscape metrics were generated using Equation (4), and are shown in Figure 4. Based on the results, the distribution trends of $C_{PLAND}$ (Figure 4a) and $C_{ED}$ (Figure 4b) exhibit similar features, where the values in the core areas are much larger than those in the peripheral areas, and the values in the east and west sides of the Pearl River Estuary obviously higher than other regions. The distribution trends of $C_{PARA\_MN}$ (Figure 4c) and $C_{AI}$ (Figure 4d) share similar features, the peripheral areas have towns with both high and low values. For example, towns in Jiangmen have relatively low values, while in Zhaoqing and Huizhou, the values are relatively high.

Based on the analyses of results, the area, shape, fragmentation degree, and aggregation state of built-up land have changed significantly from 1990 to 2015. In particular, the extent of the built-up area is increasing, the shape is becoming more complex, the level of fragmentation is becoming higher, and the aggregation degree is declining. Furthermore, the changes in landscape metrics have significant spatial distribution heterogeneity in the core and peripheral towns of cities and in the different regions of PRD. With regard to city-level changes in the built-up landscape metrics, the values of $C_{PLAND}$ and $C_{ED}$ are high in the core areas and low in the peripheral areas There are also no apparent distribution features in $C_{PARA\_MN}$ and $C_{AI}$, but with significant differences between core and peripheral areas. The reason for the differences is that the built-up lands are mainly concentrated in the central towns of cities and in the PRD’s core area. In some areas in the core towns, the proportion of built-up lands reaches 80% or higher. Thus, the growth disparity between peripheral and core areas gradually narrowed as urbanized lands expanded, forming the spatial distribution and change pattern of built-up land features in the PRD.

#### 3.1.2. Spatial Autocorrelation Analysis of Built-Up Landscape Pattern

Spatial autocorrelation analysis is used to further analyze the landscape patterns of built-up land expansion. Using the Geoda 1.14 software (GeoDa Center for Geospatial Analysis, Chicago, IL, USA), the values of Moran’s I were calculated for 1990 and 2015. The results, which are shown in Table 3, indicate that all the metrics pass the hypothesis test with the significance level of 0.05.

All the Moran’s *I* values in Table 3 are positive, which means that *L_1990_*, *L_2015_*, and *L_2015_ − L_1990_* all have positive spatial autocorrelation. Towns with high values tend to be adjacent to other towns with high values, and likewise, towns with low values are often adjacent to low-value towns. The Moran’s *I* values in *L_2015_* are larger than in *L_1990_*. The spatial autocorrelation of PARA_MN and AI increased considerably while the spatial autocorrelation of PLAND and ED remained relatively stable and only increased slightly. The Moran’s *I* values of *L_2015_ − L_1990_* are also large, indicating positive spatial autocorrelation with evident spatial agglomeration characteristics. The Moran ’s *I* values of $C_{PLAND}$ and $C_{ED}$ are significantly larger than the values of $C_{AI}$ and $C_{PARA\_MN}$.

**Figure 5:**
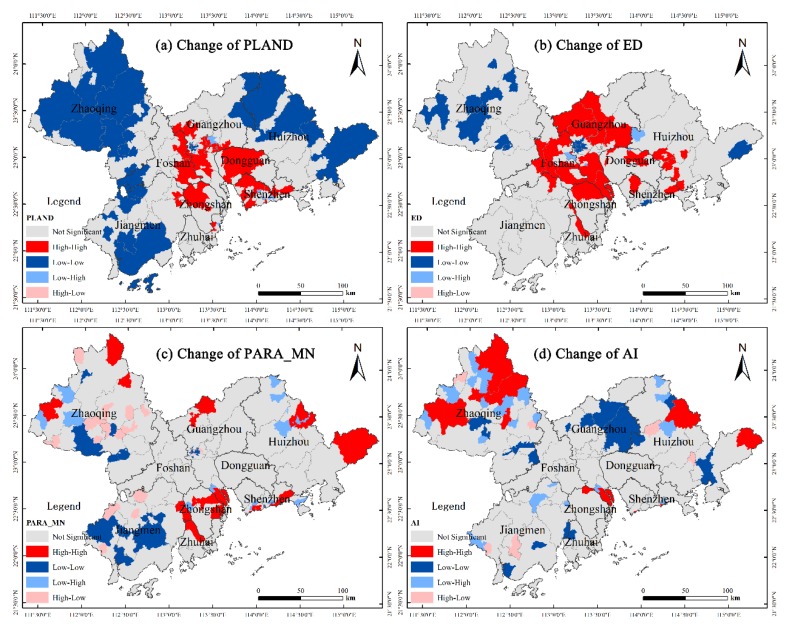
LISA Clustering map of $C_{PLAND}$, $C_{ED}$, $C_{AI}$, and $C_{PARA\_MN}$.

In order to determine spatial agglomeration areas experiencing landscape changes of built-up lands, Geoda 1.14 software (GeoDa Center for Geospatial Analysis, Chicago, IL, USA ) was used to calculate the local spatial autocorrelation indexes of $C_{PLAND}$, $C_{ED}$, $C_{AI}$, and $C_{PARA\_MN}$. After applying the LISA clustering map, non-significant types were found to be the predominant type, the High-High and Low-Low towns were numerous, and the High-Low and Low-High towns were minimal. For $C_{PLAND}$ (Figure 5a), there are 295 non-significant type areas, 114 High-High clusters concentrated around the core areas of the east and west banks of the Pearl River Estuary, 165 Low-Low areas mainly distributed in the peripheral areas of PRD (e.g., Zhaoqing, Huizhou, and Jiangmen), and six Low-High and one High-Low towns scattered mainly in the surrounding areas of High-High type towns (such as in Guangzhou and Shenzhen). For $C_{ED}$ (Figure 5b), there are 383 non-significant type areas, 96 High-High clusters distributed around the central regions of the PRD (such as in Guangzhou and Foshan), 96 Low-Low clusters concentrated in Zhaoqing, and four Low-High and two High-Low towns found mainly in Guangzhou and Foshan. For $C_{PARA\_MN}$ (Figure 5c), there are 443 non-significant type areas, 41 High-High clusters dispersed in Zhongshan, Zhuhai, Zhaoqing, and Huizhou, 59 Low-Low clusters mainly located in the cities of western PRD including Zhaoqing and Jiangmen, 14 Low-High areas in Huizhou and Zhongshan, and 24 High-Low towns situated primarily in Zhaoqing and Jiangmen. For $C_{AI}$ (Figure 5d), there are 485 non-significant type areas, 35 High-High areas distributed in northwest Zhaoqing and northeastern Huizhou, 33 Low-Low towns dispersed all over the region with a small concentration found in northern Guangzhou, 22 Low-High and six High-Low towns, and the Low-High type distribution is similar the High-High type and distributed mainly in the northwest of Zhaoqing and the northeast of Huizhou, and six High-Low towns mainly distributed in the peripheral areas of the cities of Zhaoqing, Huizhou, and Jiangmen.

The analyses show that significant spatial agglomeration exists in the landscape metrics, and the patterns in the changes of built-up lands are becoming more pronounced. The analyses on the clustering maps for $C_{PLAND}$, $C_{ED}$, $C_{AI}$, and $C_{PARA\_MN}$ show that there are more High-High and Low-Low areas compared with High-Low and Low-High towns, and there is significant spatial differentiation feature in the core and peripheral areas of the PRD.

### 3.2. Driving Forces of Built-up Land Expansion

#### 3.2.1. Potential Driving Factors

In this study, policy factors were not considered since they cannot be accurately converted into quantitative data. Meanwhile, transportation accessibility includes access to various transportation modes (e.g., highway, railway, expressway, and high-speed railway), convenience to socio-economic centers, and location advantage. This means that transportation accessibility is affected by the level of transportation development and location conditions. Based on the literature review and data availability, ten potential factors have been selected in this study namely: physical factors, including elevation (*x*_1_) and slope (*x*_2_); socio-economic factors, including per capita GDP in 1990 (*x*_3_), change in per capita GDP (*x*_4_), population density in 1990 (*x*_5_) and change in population density (*x*_6_); transportation factors, including density of road network in 1990 (*x*_7_), change in density of road network (*x*_8_), transportation accessibility in 1990 (*x*_9_), and change in transportation accessibility (*x*_10_) (Table 4). And considering that the independent variable of the geographical detector must be discrete, the method of natural breaks (Jenks) was used to divide the parameters into five classes for the geographical detector analysis.

Examining the determinants was conducted using geographical detectors, which include factor detector, interaction detector, and ecological detector. The four landscape metrics ($C_{PLAND}$, $C_{ED}$, $C_{AI}$, and $C_{PARA\_MN}$) calculated using Equation (4) were used as dependent variables while the ten impact factors were used as the independent variables for the geographical detector analysis.

#### 3.2.2. The Factor Detector

Factor detector can be used to study the forces affecting the growth of built-up land in cities. Based on the results of factor detection (summary presented in Table 5), the power (*q*) of $C_{PLAND}\;$can be ranked according to size as: *x*_6_ > *x*_2_ > *x*_5_ > *x*_1_ > *x*_4_ > *x*_8_ > *x*_10_ > *x*_7_ > *x*_9_ > *x*_3._ The explanatory powers of *x*_6_ (change in population density) and *x*_2_ (slope) are both above 0.300, which makes them primary factors affecting $C_{PLAND}$. The secondary factors (0.150 ≤ *q* ≤ 0.300) are *x*_5_, *x*_1_, *x*_4_, *x*_8_, and *x*_10_. In terms of the edge density, the ranking for *q* is as follows: *x*_2_ > *x*_7_ > *x*_5_ > *x*_8_ > *x*_6_ > *x*_10_ > *x*_4_ > *x*_9_ > *x*_1_ > *x*_3_. The primary factors (*q* > 0.200) are *x*_2_ (slope), *x*_7_ (density of road network in 1990), *x*_5_ (population density in 1990), and *x*_8_ (change in density of road network), while the secondary factors (0.150 ≤ *q* ≤ 0.200) include *x*_6_, *x*_10_ and *x*_4_. In terms of the parameter-area ratio, the ranking for *q* of $C_{PARA\_MN}$ is as follows: *x*_2_ > *x*_8_ > *x*_6_ > *x*_9_ > *x*_4_ > *x*_5_ > *x*_10_ > *x*_3_ > *x*_7_ > *x*_1_. The primary factors (*q* > 0.050) are *x*_2_ (slope), *x*_8_ (change in density of road network), and *x*_6_ (change in population density), while the secondary factors (0.030 ≤ *q* ≤ 0.050) include *x*_9_ and *x*_4_. And in terms of the aggregation index, the ranking of *q* for $C_{AI}$ is as follows *x*_1_ > *x*_2_ > *x*_9_ > *x*_5_ > *x*_4_ > *x*_3_ > *x*_6_ > *x*_7_ > *x*_8_ > *x*_10_. The primary factors (*q* > 0.100) are *x*_1_ (elevation) and *x*_2_ (slope), while the secondary factors (0.050 ≤ *q* ≤ 0.100) include *x*_9_, *x*_5_, *x*_4_ and *x*_3_.

Based on the factor detector analysis, there are distinct contrasts between the effects of factors on the change in landscape metrics of different built-up land, with $C_{PLAND}$ having the largest explanatory power and $C_{PARA\_MN}$ having the least explanatory power. Meanwhile, elevation (*x_1_*), slope (*x_2_*), population density in 1990 (*x_5_*), change in population density(*x_6_*), and density of road network in 1990 (*x_7_*) were found have strong overall explanatory powers. This shows that natural conditions (such as elevation, slope), socio-economic development (such as population density in 1990, change in population density), and transportation development level (density of road network in 1990) have vital influence on changing the landscape patterns of built-up lands. Also, societal changes can become significant driving forces for built-up land expansion.

#### 3.2.3. The Interaction Detector

The interaction detector can be used to examine interactions between factors in built-up land expansion, and there are five possible outcomes: nonlinear-weaken, uni-weaken, bi-enhance, independent and nonlinear-enhance. The results are shown in Table 6. For the 45 pairs of interactions between two factors on $C_{PLAND}$, 15 pairs are nonlinear-enhance: *x*_1_ and *x*_3_, *x*_2_ and *x*_3_, *x*_3_ and *x*_4_, *x*_3_ and *x*_5_, *x*_3_ and *x*_6_, *x*_3_ and *x*_7_, *x*_3_ and *x*_8_, *x*_3_ and *x*_9_, *x*_3_ and *x*_10_, *x*_5_ and *x*_9_, *x*_5_ and *x*_10_, *x*_7_ and *x*_8_, *x*_7_ and *x*_10_, *x*_8_ and *x*_9_, *x*_9_ and *x*_10_. The remaining 30 sets are bi-enhance. For the interactions between factors on $C_{ED}$, 16 pairs are bi-enhance: *x*_2_ and *x*_8_, *x*_3_ and *x*_5_, *x*_4_ and *x*_5_, *x*_4_ and *x*_6_, *x*_4_ and *x*_7_, *x*_4_ and *x*_8_, *x*_5_ and *x*_6_, *x*_5_ and *x*_7_, *x*_5_ and *x*_8_, *x*_5_ and *x*_10_, *x*_6_ and *x*_7_, *x*_6_ and *x*_8_, *x*_6_ and *x*_10_, *x*_7_ and *x*_8_, *x*_7_ and *x*_10_, *x*_8_ and *x*_10._ The remaining 29 sets are all nonlinear-enhance. For the interactions between factors on $C_{AI}$, 21 pairs are nonlinear-enhance, namely: *x*_1_ and *x*_4_, *x*_1_ and *x*_9_, *x*_1_ and *x*_10_, *x*_2_ and *x*_10_, *x*_3_ and *x*_4_, *x*_3_ and *x*_5_, *x*_3_ and *x*_6_, *x*_3_ and *x*_7_, *x*_3_ and *x*_8_, *x*_3_ and *x*_9_, *x*_3_ and *x*_10_, *x*_4_ and *x*_7_, *x*_4_ and *x*_9_, *x*_5_ and *x*_6_, *x*_5_ and *x*_7_, *x*_5_ and *x*_8_, *x*_5_ and *x*_10_, *x*_6_ and *x*_7_, *x*_7_ and *x*_8_, *x*_7_ and *x*_10_, *x*_9_ and *x*_10._ The remaining 24 sets are bi-enhance. For the interactions between factors on $C_{PARA\_MN}$, five pairs are bi-enhance, namely: *x*_4_ and *x*_6_, *x*_5_ and *x*_6_, *x*_6_ and *x*_8_, *x*_8_ and *x*_9_, *x*_8_ and *x*_10_. The remaining 40 sets are nonlinear-enhance.

The interaction detector reveals that the factors *x*_1_ and *x*_2_ (and more *x*) have an interactive influence on built-up land expansion. Based on the results of the interaction detector, there is a noticeable interactive influence among the different factors. There were two main types of interactions found in the study: nonlinear- enhance and bi-enhance. The combination of different factors exerts greater influence than that of a single factor. In this study, the interactions between various natural, socio-economic, and transportation-related factors have been shown to enhance the power of determinants in influencing the expansion of built-up lands. Such combinations include *x*_3_ (per capita GDP in 1990) and *x*_4_ (change in per capita GDP), *x*_5_ (population density in 1990) and *x*_6_ (change in population density), *x*_7_ (density of road network in 1990) and *x*_8_ (change in density of road network)_,_ *x*_9_ (transportation accessibility in 1990) and *x*_10_ (change in transportation accessibility). 

#### 3.2.4. The Ecological Detector

The ecological detector is used to examine the significant difference in the influence of the various factors on the four landscape metrics, is a geographical stratum more significant than another one. The results of the ecological detector analysis are shown in Table 7. For $C_{PLAND}$, there is no significant difference between *x*_1_ and *x*_4_, *x*_1_ and *x*_5_, and *x*_1_ and *x*_8_. There are significant differences between *x*_2_ and other factors. There is no significant difference between *x*_3_ and *x*_7,_ *x*_3_ and *x*_9_, *x*_4_ and *x*_5_, *x*_4_ and *x*_8,_ *x*_4_ and *x*_10,_ and *x*_5_ and *x*_8_. There are significant differences between *x*_6_ and other factors. There is no significant difference between *x*_7_ and *x*_8_, *x*_7_ and *x*_9_, *x*_7_ and *x*_10_, *x*_8_ and *x*_10_, and *x*_9_ and *x*_10_. For $C_{ED}$, there no significant difference between *x*_1_ and *x*_3_, *x*_1_ and *x*_4_, *x*_1_ and *x*_9_, *x*_2_ and *x*_5_, *x*_2_ and *x*_6_, *x*_2_ and *x*_7_, *x*_2_ and *x*_8_, *x*_3_ and *x*_9_, *x*_4_ and *x*_5_, *x*_4_ and *x*_6_, *x*_4_ and *x*_7_, *x*_4_ and *x*_8_, *x*_4_ and *x*_9_, *x*_4_ and *x*_10_; *x*_5_ and *x*_6_, *x*_5_ and *x*_7_, *x*_5_ and *x*_8_, *x*_5_ and *x*_10_,*x*_6_ and *x*_7_, *x*_6_ and *x*_8_, *x*_6_ and *x*_10_, *x*_7_ and *x*_8_, *x*_7_ and *x*_10_; *x*_8_ and *x*_10_, and *x*_9_ and *x*_10_. For $C_{PARA\_MN}$, there is no significant difference among factors. For $C_{AI}$, there is no significant difference among the factors except for the pairs: *x*_1_ and *x*_7_, *x*_1_ and *x*_8_, *x*_1_ and *x*_10_, and *x*_2_ and *x*_10._

In the ecological detector, results of the statistically significant differences between two factors are presented. If Y(row) was significantly bigger than Y(column), the associated value is “Y”, while “N” expresses the opposite meaning. The results show there are significant differences in the influence of determinants for the different landscape metrics. However, there is no significant difference in the influence of factors on $C_{PARA\_MN}$ and $C_{AI}$. For $C_{PLAND}$ and $C_{ED}$, a considerable number of pairings were shown to have significant differences. In particular, the ecological detector results of $C_{PLAND}$ shows that there are significant differences between *x*_2_ and all the other factors and between *x*_6_ and all the other factors. This suggests that slope (*x*_2_) and change in population density (*x*_6_) have unique roles in influencing the expansion of built-up lands.

### 3.3. Limitations

There are several limitations to this study. First, we chose to focus our analysis only on the years 1990 and 2015, and we did not include intervening years to check short-term temporal trends. Future studies can utilize long-term and higher temporal scale datasets as improvements. Second, a limited set of physical, socio-economic and transportation-related determinants were utilized in the geographical detector analysis. Other variables, such as policy factors, may be considered in future studies. Third, the geographical detector model can only probe the interactive influences between two driving factors. Different approaches and methodological modifications can be proposed and developed in the future in order to analyze the compounding and complex interactive influences of three or more driving factors.

## 4. Conclusions

Against the backdrop of rapid urbanization, this study analyzes built-up land expansion in PRD for 1990 and 2015 using Landsat images. Using four landscape metrics, percentage of landscape, edge density, aggregation index, and perimeter-area ratio_mean, to indicate built-up land expansion features, an extensive assessment of the landscape patterns of built-up land expansion was conducted that combines global and local spatial autocorrelation techniques. Further analyses were employed evaluating determinants of built-up land expansion using geographical detectors that included factor detector, interaction detector, and ecological detector. The key findings can be summarized as follows:

First, during the 25 years (1990–2015), there had been significant changes in the area, shape, fragmentation degree, and aggregation degree of built-up lands in PRD. In particular, the urban area has increased in size, the shape has become more complex, the level of fragmentation degree has become higher, and the aggregation degree has been in decline. With the rapid development of economy and population, the increase in human activities has led to dramatic changes in the scale and pattern of built-up lands in PRD.Second, in calculating the data involving the 581 towns in PRD, the built-up landscape metrics exhibit significant spatial distribution differences in the core and peripheral towns of each city, as well as the core and peripheral areas of the PRD. The analyses show that significant spatial agglomeration exists in the landscape metrics, and the patterns in the changes of built-up lands are becoming more pronounced. Regarding $C_{PLAND}$ and $C_{ED}$, the core areas were shown to have high values while the peripheral areas have low values. For the $C_{AI}$ and $C_{PARA\_MN}$, there are no distinct patterns of value concentrations, but the core and peripheral areas still exhibited significant differences. In terms of the spatial agglomeration of areas experiencing landscape changes, the High-High and Low-Low towns were much greater in number than High-Low and Low-High towns, which indicates clear spatial differences in the core and peripheral regions of the PRD.

Finally, this study proves that the geographical detector can be an effective method for analyzing the driving forces influencing built-up land expansion. The results of the factor detector show that natural, socio-economic, and transportation-related determinants have played essential roles in the growth of built-up lands. In particular, the overall explanatory powers of elevation (*x*_1_), slope (*x*_2_), population density (*x*_5_), change in population density (*x*_6_), and road network density (*x*_7_) were found to have strong influence on urban land expansion. The results of the interaction detector show that there are relationships among the different factors, including enhance non-linear and enhance bivariate interactions. This means the interaction between different factors enhances the power of determinants in influencing the expansion of build-up lands. The results of the ecological detector show that the influencing powers of factors on $C_{PARA\_MN}$ and $C_{AI}$ have no significant differences, while the powers of determinants on $C_{PLAND}$ and $C_{ED}$ have significant differences. In particular, the ecological detector results of $C_{PLAND}$ show that there are significant differences between slope (*x*_2_) and the other factors and between population density change (*x*_6_) and other factors, which indicate that slope and changes in population density have significant impact on the expansion of built-up lands. The factor detector, interaction detector and ecological detector can identify the driving forces of urban land growth and their interactions, can provide reference for other research about driving forces.

## Figures and Tables

**Table 1:** Landscape metrics used in the study.

Metrics	Acronym	Description
Percentage of landscape	PLAND	PLAND indicates the proportion of the built-up land patch(class) over the entire landscape area; the value of PLAND increases with greater urbanization and built-up land expansion.
Edge density	ED	ED indicates the ratio of the total edge length of all built-up land patches over the total landscape area. Larger values mean higher fragmentation.
Aggregation Index	AI	AI is calculated using the adjacency matrix. It provides information about specific aspects of landscape composition and structure caused by urbanization and helps characterize urbanization in terms of stability. Higher values mean greater clustering.
Perimeter-Area Ratio_Mean	PARA_MN	PARA is a simple measure of shape complexity and is equal to the ratio of the built-up land patch perimeter and the area, an increase in patch size will cause a decrease in the perimeter-area ratio. PARA_MN indicates the mean of PARA.

**Table 2:** Built-up landscape metrics.

	1990	2015
PLAND	2.21	16.69
ED	2.31	31.07
PARA_MN	687.08	1048.36
AI	91.37	86.04

**Table 3:** Statistical on Moran’s *I.*

	*L*_1990_	*L*_2015_	*L_2015_ − L_1990_*
PLAND	0.845	0.859	0.689
ED	0.615	0.685	0.644
PARA_MN	0.302	0.666	0.285
AI	0.436	0.801	0.243

**Table 4:** Ten potential factors selected in this study.

Factors	Description
*x*_1_	elevation
*x*_2_	slope
*x*_3_	per capita Gross Domestic Product (GDP) in 1990
*x*_4_	change in per capita GDP
*x*_5_	population density in 1990
*x*_6_	change in population density
*x*_7_	density of road network in 1990
*x*_8_	change in density of road network
*x*_9_	transportation accessibility in 1990
*x*_10_	change in transportation accessibility

**Table 5:** Factor detector of the geographical detector.

	*x*_1_	*x*_2_	*x*_3_	*x*_4_	*x*_5_	*x*_6_	*x*_7_	*x*_8_	*x*_9_	*x*_10_
*C_PLAND_*	0.250	0.394	0.039	0.229	0.256	0.406	0.134	0.185	0.082	0.155
*C_ED_*	0.077	0.258	0.051	0.168	0.211	0.182	0.214	0.209	0.085	0.174
*C_PARA_MN_*	0.015	0.058	0.019	0.032	0.026	0.055	0.016	0.056	0.048	0.025
*C_AI_*	0.135	0.127	0.053	0.055	0.062	0.042	0.034	0.031	0.082	0.023

**Table 6:** Interaction detector of the geographical detector.

		*x*_1_	*x*_2_	*x*_3_	*x*_4_	*x*_5_	*x*_6_	*x*_7_	*x*_8_	*x*_9_			*x*_1_	*x*_2_	*x*_3_	*x*_4_	*x*_5_	*x*_6_	*x*_7_	*x*_8_	*x*_9_
PLAND	*x*_2_	0.41									PARA_MN	*x*_2_	0.10								
	*x*_3_	0.35	0.48									*x*_3_	0.10	0.13							
	*x*_4_	0.39	0.50	0.38								*x*_4_	0.12	0.16	0.08						
	*x*_5_	0.39	0.53	0.45	0.37							*x*_5_	0.09	0.13	0.07	0.07					
	*x*_6_	0.50	0.63	0.49	0.48	0.49						*x*_6_	0.10	0.15	0.08	0.08	0.08				
	*x*_7_	0.33	0.45	0.27	0.34	0.37	0.53					*x*_7_	0.07	0.11	0.08	0.12	0.12	0.13			
	*x*_8_	0.35	0.45	0.33	0.37	0.38	0.49	0.34				*x*_8_	0.09	0.12	0.09	0.10	0.09	0.11	0.11		
	*x*_9_	0.29	0.45	0.21	0.31	0.36	0.49	0.20	0.32			*x*_9_	0.12	0.15	0.09	0.11	0.10	0.13	0.11	0.11	
	*x*_10_	0.31	0.46	0.35	0.38	0.42	0.50	0.36	0.30	0.37		*x*_10_	0.07	0.1	0.09	0.08	0.09	0.12	0.12	0.07	0.12
ED	*x*_2_	0.35									AI	*x*_2_	0.17								
	*x*_3_	0.21	0.37									*x*_3_	0.17	0.18							
	*x*_4_	0.33	0.45	0.28								*x*_4_	0.20	0.18	0.12						
	*x*_5_	0.36	0.49	0.26	0.28							*x*_5_	0.17	0.18	0.13	0.10					
	*x*_6_	0.34	0.47	0.29	0.27	0.29						*x*_6_	0.15	0.15	0.11	0.09	0.11				
	*x*_7_	0.32	0.48	0.32	0.36	0.38	0.38					*x*_7_	0.17	0.16	0.12	0.13	0.14	0.10			
	*x*_8_	0.30	0.42	0.28	0.31	0.33	0.3	0.38				*x*_8_	0.15	0.14	0.11	0.09	0.10	0.06	0.10		
	*x*_9_	0.23	0.35	0.21	0.30	0.32	0.3	0.34	0.33			*x*_9_	0.22	0.19	0.14	0.15	0.13	0.12	0.10	0.1	
	*x*_10_	0.31	0.45	0.29	0.35	0.37	0.35	0.36	0.28	0.32		*x*_10_	0.18	0.1	0.11	0.08	0.10	0.06	0.11	0.05	0.15

**Table 7:** Ecological detector of the geographical detector.

		*x*_1_	*x*_2_	*x*_3_	*x*_4_	*x*_5_	*x*_6_	*x*_7_	*x*_8_	*x*_9_			*x*_1_	*x*_2_	*x*_3_	*x*_4_	*x*_5_	*x*_6_	*x*_7_	*x*_8_	*x*_9_
PLAND	*x*_2_	Y									PARA_MN	*x*_2_	N								
	*x*_3_	Y	Y									*x*_3_	N	N							
	*x*_4_	N	Y	Y								*x*_4_	N	N	N						
	*x*_5_	N	Y	Y	N							*x*_5_	N	N	N	N					
	*x*_6_	Y	N	Y	Y	Y						*x*_6_	N	N	N	N	N				
	*x*_7_	Y	Y	N	Y	Y	Y					*x*_7_	N	N	N	N	N	N			
	*x*_8_	N	Y	Y	N	N	Y	N				*x*_8_	N	N	N	N	N	N	N		
	*x*_9_	Y	Y	N	Y	Y	Y	N	Y			*x*_9_	N	N	N	N	N	N	N	N	
	*x*_10_	Y	Y	Y	N	Y	Y	N	N	N		*x*_10_	N	N	N	N	N	N	N	N	N
ED	*x*_2_	Y									AI	*x*_2_	N								
	*x*_3_	N	Y									*x*_3_	N	N							
	*x*_4_	N	Y	Y								*x*_4_	N	N	N						
	*x*_5_	Y	N	Y	N							*x*_5_	N	N	N	N					
	*x*_6_	Y	N	Y	N	N						*x*_6_	N	N	N	N	N				
	*x*_7_	Y	N	Y	N	N	N					*x*_7_	Y	N	N	N	N	N			
	*x*_8_	Y	N	Y	N	N	N	N				*x*_8_	Y	N	N	N	N	N	N		
	*x*_9_	N	Y	N	N	Y	Y	Y	Y			*x*_9_	N	N	N	N	N	N	N	N	
	*x*_10_	Y	Y	Y	N	N	N	N	N	N		*x*_10_	Y	Y	N	N	N	N	N	N	N

F test with a significance level of 0.05 is used and Y indicates that there is a significant difference in the influence of two factors on the landscape pattern, while N indicates that there is no significant difference.
